# Migration corridors in Africa and access to health services: Current challenges and a path forward for research and practice

**DOI:** 10.4102/jphia.v17i1.1626

**Published:** 2026-02-13

**Authors:** Edward Kirumira, Muhammad H. Zaman, Helen E. Lindsay, Julia Pettengill

**Affiliations:** 1 Stellenbosch Institute for Advanced Study, Stellenbosch, South Africa; 2 Center on Forced Displacement, Boston University, Boston, United States of America; 3 The Schooner Foundation, Boston, United States of America

**Keywords:** migration, migration corridors, health services access, forced displacement, mental health

## Abstract

This commentary discusses migration corridors on the African continent and access to health services. It stems from a workshop on migration corridors held in South Africa and reflects the interdisciplinary collaborative dialogue on migration journeys and healthcare, incorporating physical and mental well-being. We must reimagine migration narratives and healthcare accessibility and call for new methods of knowledge generation and service provision. By framing the migration journey as corridors that take many directions, beyond the ‘Global South’ to ‘Global North’ paradigm, we propose that the healthcare sector can more effectively utilise interdisciplinary research methods centring the well-being of migrants. Considering this reframing, we call for reimagined funding structures, ethical technology use and new methods of knowledge generation and service provision.

## Introduction

As scholars and practitioners working in the intersection of health and migration, in Africa as well as globally, we call attention to the critical need to reimagine how researchers and practitioners work together, especially given the changing geopolitical context impacting the humanitarian sector. In this commentary, we share our perspective on key issues and ideas for sustainable, ethical and effective collaboration, stemming from our workshop held at the Stellenbosch Institute. The issue of migration has become one of global significance, as over 120 million people are forcibly displaced from their homes.^1^ Displacement owing to new and protracted armed conflicts in several regions, combined with increasing impacts of climate change, extremism, xenophobia and economic collapse of the state, deserves serious and sustained attention.^1^ Migration is associated with health risks and health inequities in multiple regions.^2^ Public health challenges associated with migration include mental health impacts of forced displacement, spread of infectious disease in conflict-affected regions or along journeys, interrupted chronic disease treatment and inaccessibility of healthcare in transit or host countries.^2^ For example, the World Health Organization (WHO) reports that globally, refugee and migrant groups were disproportionately impacted by coronavirus disease 2019 (COVID-19).^3^ Only 23.1% of South Sudanese refugees in Uganda’s Rhino Camp had access to adequate handwashing facilities.^3^ In Durban, South Africa, 92% of female refugees face food insecurity, as do 33% of migrants in Libya, contributing to poorer health outcomes.^3^

Forcibly displaced persons often migrate through several countries on their journey to safety; these paths – traversing countries and regions – are broadly referred to as migration corridors.^4^ While movement across individual borders from one country to another has been studied and reported on, migration corridors have received inadequate attention in academic literature and in news media. Migration corridors are important in Africa as well but have only gotten limited attention in non-governmental organisation (NGO) or academic sectors. Forcibly displaced persons in Africa are moving not just to the European Union (EU) but also south on the continent.^4^ Issues around access to healthcare and social determinants of health, such as housing and safety, remain largely unexplored despite their enormous significance for human dignity and well-being.^5^

Given the urgency of the topic, the need for interdisciplinary engagement and limited attention to migration corridors in Africa, the Center on Forced Displacement at Boston University, the Stellenbosch Institute for Advanced Study and the Schooner Foundation held a 2-day workshop focused on healthcare access along migration corridors in Africa centred on the question of how to think about these issues collaboratively and inclusively, with scholars, community workers, care providers, to generate knowledge in ethical ways and support migrants effectively.

## Rethinking migration narratives

The media too often centres migration corridors from ‘South to North’, creating a false narrative that all migrants from the African continent are attempting to reach Europe.^6,7^ Our conversation challenges dominant migration narratives of the continent, considering not only migration to Europe but also corridors within the continent or to the Middle East. In political discourse, there is a weaponisation of the migration narrative, which endangers migrants. Research has shown that narratives that exacerbate stigma and discrimination against migrants contribute to worse health outcomes for this group by exacerbating barriers to healthcare access and increasing mental health issues.^8,9^ We need to be communicating in a way that responsibly demonstrates the diversity and complexity of migration experiences, serving to reduce stigma against migrants.

Currently, states use corridors to control migration, but the utility of corridors as a tool for understanding and working for activists is less discussed, while migrants’ experience of them as sites of vulnerability or resilience is not part of the conversation. We must reimagine how we view corridors as pathways to support healthcare delivery and activism, to amplify migrants’ personal agency and support reintegration for those who wish to return to their country of origin. To do this, however, we must interrogate the (often state-defined) legal-vs-illegal and formal-vs-informal binaries to understand the moral and political dimensions of illegality that can exacerbate vulnerability and the impact on accessibility of services.

The narrative of migration corridors is, of course, not just about the locations within the corridors; the individuals who travel the corridors should be at the centre of the narrative. The term ‘migrant’, too often framed with a negative connotation, must be reimagined in three primary ways. Firstly, migrants are not exclusively ‘the other’ as potentially anyone may become a migrant if their circumstances change due to political instability, violence or natural disaster. Secondly, migrant is not a permanent status, while some remain forcibly displaced their whole lives, many have and can move in and out of that status and/or definition, whether through resettlement or return to their place of origin. Thirdly, migrant is an umbrella term, often used to refer to different categories of forced displacement and movement from one’s place of residence.^10^

## Healthcare access and information

The interdisciplinary discussion brought together community workers, medical providers, social workers and scholars from public health, policy and social science engaged in thinking deeply about both physical and mental well-being along migration corridors. Broadly, healthcare needs of migrants often go unaddressed, and particular segments of the population, such as informal migrants or marginalised populations, often do not receive care or receive inadequate care.^6^

In South Africa, the WHO reports that in areas with higher numbers of migrants, participation in antenatal care is lower, and in Uganda, refugee women report being discriminated against and receiving unsatisfactory care at antenatal visits.^3^ Non-governmental organisation partners in the workshop underscored the importance of Mental Health and Psychosocial Support (MHPSS) in displacement situations. In emergency situations, this support is not available enough and is inadequate to support the needs of the population. Unfortunately, it is not widely addressed in philanthropy or policy but is crucial to migration narratives.

Equipping healthcare providers with knowledge and tools is critical. Interdisciplinary thinking allows us to reimagine how to do this. Medical professionals in cross-border migration settings and fragile scenarios have expressed a need for health histories when treating patients.^11^ While technology may be able to bridge this gap, community-informed perspectives identify the need for safe platforms for sharing medical histories that protect the privacy and dignity of patients.^12^ Translation too can be a critical need for medical providers and community workers; here too, technology is an option but must be used safely and ethically. Yet an interdisciplinary approach underscores the importance of the humanities. Art can be a useful tool in helping individuals faced with severe illness and a method of expression that can be supportive when used in MHPSS.

Addressing health needs must prioritise locally led solutions, and when partnerships are established, they should focus on trust-based, long-term support. Community-based partners emphasised this in the workshop; in order to have health equity along migration corridors, we need to resource the margins. Humanitarian and development aid is not reaching those who need it most as it is too often donor-driven priorities, which receive the most attention. To effectively support locally- led solutions, funders must shift focus to funders to resource this work differently. Funding priorities should be responsive to local priorities, focusing on multi-year efforts, unrestricted funding and systems-focused approaches.^13^ Critically, steps must be taken to avoid extractive models and to instead be accountable to the community, open to learning during the projects.^13^

## New ways of knowledge generation and service provision: Bridging research, practice and activism

In rethinking how we disseminate accurate and representative migration narratives and in how to provide effective care that reaches the most vulnerable, we see the necessity of bridging research, practice and activism and bringing the knowledge and skills of this bridging into educational settings.

To build this bridge, researchers, alongside community organisations, should employ participatory research methods, involving community members in identifying priority areas for research, co-developing the research process and empowering community members through sharing results.^14^ We must reimagine how we produce knowledge and place emphasis on co-produced knowledge and emancipatory methods. In our workshop, which included artists and community workers, we discussed body mapping (visual representations of the body used to express emotions and experiences) and storytelling as methods that can be used for empathetic communication around sensitive topics and allow research participants to express themselves in new ways. When employing these and other methods, we must avoid extractive research and focus on building sustainable, reciprocal research partnerships with communities and NGOs. To do this, we need effective conceptual tools to integrate theory and action across disciplines and practical tools to support partnerships between academic and community organisations. It will also require us to reimagine how we meet; moving from one-off conferences to sustained, cross-disciplinary collaborations, regularly convening in spaces where listening, co-creation and sharing replace traditional academic presentations.

As we do this, we must prepare the next generation of scholars and practitioners to work sustainably and collaboratively in this field. Currently, migration knowledge is not filtering into higher education pedagogy effectively, and professionals engaged in working with migrant populations, such as teachers and healthcare workers (HCWs), feel unprepared.^15,16,17^ Migration topics should be embedded in fieldwork and courses. Teaching empathy, especially in disciplines like social work, is vital for shaping future practitioners. Some of the same tools previously discussed, like body mapping, could be useful here. As this is a globally relevant topic, it is critical that knowledge be made accessible to those working in the field; online courses and short programmes can be tools for scaling knowledge and connecting NGOs and academia. Migration research should draw lessons from public health, human immunodeficiency virus (HIV) advocacy and other sectors to effectively build this bridge between research, advocacy and practice.

## Conclusion

This commentary explored migration corridors on the African continent through three themes: narratives, health access and information and new ways of research and service provision. We support the framing of the migration journey as a corridor and encourage an honest portrayal of migration narratives, as moving in many directions, beyond the ‘Global South’ to ‘Global North’ paradigm. In the healthcare sector, we promote interdisciplinary, holistic approaches that focus not solely on the absence of disease but also on physical and mental well-being and dignity. We call for updated methods of funding that are multi-year and unrestricted and for technology to be used in ethical ways that prioritise the safety and privacy of displaced persons. Lastly, we present ideas for new ways of knowledge generation and service provision that centre communities’ voices, avoid extractive methods and build sustainable partnerships, calling for education that trains young scholars and practitioners on these approaches.
